# Gallic Acid Content and an Antioxidant Mechanism Are Responsible for the Antiproliferative Activity of ‘Ataulfo’ Mango Peel on LS180 Cells

**DOI:** 10.3390/molecules23030695

**Published:** 2018-03-19

**Authors:** Gustavo. R. Velderrain-Rodríguez, Heriberto Torres-Moreno, Mónica A. Villegas-Ochoa, J. Fernando Ayala-Zavala, Ramón E. Robles-Zepeda, Abraham Wall-Medrano, Gustavo A. González-Aguilar

**Affiliations:** 1 Coordination of Food Technology of Plant Origin, Center for Research in Food and Development, A.C. (CIAD), Carretera a la Victoria Km 0.6. C.P., Hermosillo 83304, Mexico; grvelderrain@gmail.com (G.R.V.-R.); mvillegas@ciad.mx (M.A.V.-O.); jayala@ciad.mc (J.F.A.-Z.); 2 Department of Biological Chemistry., Universidad de Sonora, Blvd. Luis Encinas y Rosales S/N Col. Centro, C.P., Hermosillo 83000, Mexico; heriberto.torres@unison.mx (H.T.-M.); rrobles@guayacan.uson.mx (R.E.R.-Z.); 3 Biomedical Sciences Institute, Autonomous University of Ciudad Juarez, Anillo Envolvente del Pronaf y Estocolmo S/N, Ciudad Juárez 32310, Chihuahua, Mexico; awall@uacj.mx

**Keywords:** phenolic compounds, by-products, biological activity, LS180, colon cancer, antioxidant mechanism, single electron transfer (SET), hydrogen atom transfer (HAT)

## Abstract

Mango “Ataulfo” peel is a rich source of polyphenols (PP), with antioxidant and anti-cancer properties; however, it is unknown whether such antiproliferative activity is related to PP’s antioxidant activity. The content (HPLC-DAD), antioxidant (DPPH, FRAP, ORAC), and antiproliferative activities (MTT) of free (FP) and chemically-released PP from mango ‘Ataulfo’ peel after alkaline (AKP) and acid (AP) hydrolysis, were evaluated. AKP fraction was higher (µg/g DW) in gallic acid (GA; 23,816 ± 284) than AP (5610 ± 8) of FR (not detected) fractions. AKP fraction and GA showed the highest antioxidant activity (DPPH/FRAP/ORAC) and GA’s antioxidant activity follows a single electron transfer (SET) mechanism. AKP and GA also showed the best antiproliferative activity against human colon adenocarcinoma cells (LS180; IC_50_ (µg/mL) 138.2 ± 2.5 and 45.7 ± 5.2) and mouse connective cells (L929; 93.5 ± 7.7 and 65.3 ± 1.2); Cheminformatics confirmed the hydrophilic nature (LogP, 0.6) and a good absorption capacity (75%) for GA. Data suggests that GA’s antiproliferative activity appears to be related to its antioxidant mechanism, although other mechanisms after its absorption could also be involved.

## 1. Introduction

Mango (*Mangifera indica* L.) cv. ‘Ataulfo’ is potentially the most important Mexican mango cultivar of nutraceutical interest. Mexico is the leading global exporter of mangoes, and the ‘Ataulfo’, ‘Tommy Atkins’, ‘Hayden’, and ‘Kent’ varieties account for 60% of the mangoes produced nationally [1,2]. Mango pulp is usually sold fresh-cute, canned, processed as juices, nectars, jams, sun dried, and even freeze-dried [3,4,5]. However, more than 50% of the bio-waste generated by the mango agroindustry (seeds (~14%), pomace (~18%), and peel (~18%)) can be a source of several phytochemicals, such as polyphenols (PP), with health benefits (e.g., anti-cancer effects) and useful food-related properties that can be used as ingredients in other foods [6,7].

Previously identified free-polyphenols (FP) species within aqueous ‘Ataulfo’ peel extracts are xanthones (mangiferin), flavonoids (epicatechin and catechin), and phenolic acids (gallic, chlorogenic, protocatechuic, and syringic acids) [8]. These compounds may have synergistic, additive or antagonistic antioxidant activities due to several molecular polyphenol–polyphenol interactions or food matrix association [9,10,11]. The bioavailability of PP is usually low, as they have diverse possible interactions with either food matrix or other biomolecules, varying their pharmacokinetics according to food composition [12]. In this sense, polymeric forms of mango peel’s PP (e.g., gallotannins) are bounded to both insoluble and soluble dietary fibers (a.k.a. “antioxidant fibers”) which reduces the bioaccessibility of the PP fraction under simulated gastrointestinal conditions [13,14,15]. However, from an industrial scale-up perspective, chemical (e.g., alkaline and acid) or enzymatic (e.g., tanasse) hydrolysis of these complex phenolics are more common ways by which monomeric (from complex PP) or aglycones (from sugar-bounded PP) can be recovered. In this sense, many nutraceutical preparations could be prepared using otherwise discarded mango peels or other agro industrial byproducts from fruits [16,17,18].

Many studies have confirmed the anticancer potential of mango pulp and its byproducts. Noratto, et al. [19] and Corrales-Bernal, et al. [20] have reported that FP extracts from “Keith” mango pulp had chemotherapeutic potential against breast cancer, being in part involved in the PI3K/AKT pathway and miR-126. Matkowski, et al. [21] reported that ‘Ataulfo’ and ‘Haden’ mango pulps have higher antioxidant and antiproliferative effects on colon cancer cells (SW-480) compared to other varieties. Gold-Smith, et al. [22] observed an antiproliferative effect of ‘Azúcar’ mango on SW-480 cells and a beneficial effect in a rodent model of colorectal cancer after 10 weeks of treatment. However, this type of study usually underestimates the individual role and contribution of macromolecular antioxidants which are even richer in PP monomers that, under normal physiological conditions, represent the non-bioaccessible fraction (at small intestine) reaching the colon to exert their potential benefits (e.g., antiproliferative effect) after microbial fermentation of dietary fibers.

Thus, the aim of this study was to quantify the amount of free (FP) and monomeric PP released after alkaline (AKP) or acid (AP) hydrolysis from mango cv. ‘Ataulfo’ peel, as well as the antioxidant capacity and antiproliferative activity in colon cancer cells (LS180 (ATCC^®^ CL-187™)) of FP, AKP, and AP.

## 2. Results and Discussion

### 2.1. Free and Bound Polyphenols from ‘Ataulfo’ Mango Peel

Table 1 shows the content of monomeric polyphenols in the different extracts (FP, AKP, and AP) obtained from ‘Ataulfo’ mango peel. Mangiferin was found in all fractions (µg/g): FP (1259; 53%) > AKP (967; 40%) > AP (157; 7%). This xanthone is present in leaves, stems, bark, and fruits of the mango tree, but it is also widely distributed in other plants from the Anacardiaceae and Gentianaceae families [23,24]. Mangiferin is a heat-stable molecule, and, unlike more common *O*-glycosides, it is relatively resistant to hydrolysis of its aglycone (noratiriol) and sugar moieties, which explains why it was detected even after alkaline and acid hydrolysis in the present study. However, the exposure of free mangiferin to 0.1 M NaOH for 3 h leads to its fragmentation, so the stronger alkaline conditions used in our study may have also resulted in a substantial loss of mangiferin, particularly in the AKP fraction [25]. Considering total mangiferin content as the sum of amounts in the three mango peel extract’s fractions, FP showed 53% of mangiferin, indicating that its presence within mango peel is mainly as a free non-bound aglycone molecule. Ma, et al. [26] isolated mangiferin from ‘Chaunsa’ mango peel through a solvent partition method using petroleum ether (to remove fatty matter), cold acetone (to separate tannins), and 70% ethanol (to isolate mangiferin), obtaining a higher mangiferin content than has been seen in other mango varieties (“Anwar ratol”, “Langra”, “Dusahri”, and “Desi”). In contrast, Xiao, et al. [27] reported that the highest contents of FP and mangiferin from ‘Chaunsa’ mango peel were obtained using 80% methanol or ethanol (~60 mg GAE; 100–110 µg/g) instead of 80% acetone (~30 mg GAE) or ethyl acetate (~20 mg GAE). However, since mangiferin molecule is insoluble in both hydrophilic and hydrophobic media it should be complexed with other molecules such as phospholipids or polycaprolactone/poly(ethyleneglycol)/polycaprolactone (PCEC) microspheres to enhance its solubility, food release, bioavailability, and other properties [11,12].

Gallic acid (GA) was the most abundant molecule in mango peel, however, it is not found in its aglycone form but bound to its food matrix or as its polymeric form known as gallotanins. Acosta-Estrada, et al. [28] used MALDI-TOF/TOF MS to identify a diverse range of gallotannins from penta- to trideca-*O*-galloylglucose that were obtained from defatted mango ‘Ataulfo’ peel, extracted with 50% acidified-methanol (pH 2.0) at room temperature for 1 h. Sun and Cheng [29] reported that more restricted sizes of gallotanins (penta- to nona-*O*-galloyl-glucosides) could be extracted from three Chinese mango cultivars with 80% acetone. The prolonged (overnight) stepwise alkaline and acid treatments used in this study seem to be more effective for hydrolyzing gallotannins into monomeric GA residues because, as mentioned by Barnes, et al. [30], alkaline hydrolysis results in smaller losses of polyphenols compared to high temperature acid hydrolysis. The high extraction efficiency of alkaline hydrolysis is due to a mechanism involving the saponification of the ester bonds that serve as the crosslinkers between the xylan hemicelluloses, and components such as lignins and other hemicelluloses [31]. Mattila and Kumpulainen [32] reported that an in vitro hydrolysis of gallotannins from ‘Keitt’ mango decreased their relative composition of galloyl derivatives, which serve as a pool of GA to be either absorbed or metabolized by the gut microbiome. This situation may result in the systemic distribution of GA and enhance its anti-cancer potential in different cells [33]. It should be noted that by reducing the time of exposure from 16 to 8 h, as previously suggested by Razzaghi-Asl, et al. [34], the degradation of GA in the AKP fraction was minimized. Whether protocatechuic acid, which is only detected in AKP, was a product of the partial dehydroxylation (at the 5th position) of GA or if it was from a preexisting fragment is unknown.

Many other PP species were found in low to moderate concentrations (12–700 µg/g), such as quercetin, catechin, rutin, and *p*-coumaric, 2-hydroxybenzoic, protocatechuic, ferulic, syringic, and ellagic acids. Jakobek [35] also identified mangiferin, catechin, and quercetin in peels of different mango cultivars extracted with 80% acetone. Hydroxycinnamic acids are commonly found in plants in their esterified and glycosylated forms, as complexed derivatives such as dimers and trimers or as mixed glycosides; however, most hydroxybenzoic acids are commonly bound to fibers, sugars, or proteins by hydrogen bonding or other weak interactions [6,36]. The PP profile reported here was quite similar to that of the 80% acetone extract of ‘Badami’ mango peel (raw & ripe) in which most species were found as their glycosylated derivatives [8]. Thus, our results suggest that AKP fraction contains higher monomeric aglycone species, especially GA, released after PP depolymerization or food matrix ester linkage hydrolysis.

### 2.2. Antioxidant Activity of Mango “Ataulfo” Peel Polyphenols

The comprehensive evaluation of the antioxidant activities of monomer aglycone PP standards and the extract’s fractions (FP, AKP, and AP) comprised three different methods in order to capture the most common mechanisms: single electron transfer (SET) and hydrogen atom transfer (HAT). According to data shown in Table 2, AKP and AP fractions had higher antioxidant activities, with the AKP fraction being more effective in DPPH/FRAP/ORAC assays. Previous reports indicate that at least in the case of phenolic acids, combined antioxidant effects could be observed due to their molecular interactions [21,37].

Even when Cos, et al. [38] reported that mangiferin usually has comparable or higher activity than other PP in the DPPH assay, in this study the most potent DPPH radical scavengers (IC_50_) were GA, ellagic acid, quercetin, and catechin (around 0.01 µmoles of antioxidant/mL). We have previously reported that GA (acting by a SET mechanism) is a better DPPH scavenger than protocatechuic acid (also via a SET mechanism) in a synergistic manner, which confirms the result presented here [9]. The superiority of GA as an antioxidant compared to other PP, such as quercetin and rutin, is also supported by other reports [11,39,40]. However, our results suggest that GA higher activity is by its easiness to act mainly through SET mechanism, as it has the highest values in FRAP, and the lowest in ORAC assays. As described previously, AKP fraction has the higher amount of GA; however, it represents only approximately 2.38% of the dry weight of AKP fraction. However, these results do not consider possible synergistic or antagonistic effects that may be occurring.

Additionally, antioxidant activity by DPPH assay for penta-*O*-galloyl-glucoside (IC_50_ = 1.2 μg/mL) was lower than GA (IC_50_ = 0.7 μg/mL), as reported by Marino, et al. [41]. These results support the fact that AKP was higher than FP antioxidant activity, as it is the fraction with higher amounts of this molecule. In agreement, comparison between these results suggest that when GA is in its polymeric form, it could reduce its ability to transfer electrons when acting as an antioxidant. This possibility was suggested because the levels of free GA in FP and AP were the lowest, whereas AKP (higher levels of free GA) had higher antioxidant values in all methods of this study. Interestingly, AKP was a more potent antiproliferative extract than other free PP or extract’s fractions assayed, which will be further discussed. In contrast, Palafox-Carlos, Gil-Chávez, Sotelo-Mundo, Namiesnik, Gorinstein, and González-Aguilar [8] reported that all three aromatic hydroxyl groups of GA are prone to oxidation under extreme alkaline conditions and produce hydrogen peroxide, quinones, and semiquinones as degradation products; in fact, only deprotonated (~80%) and mono-protonated (~20%) GA exists above pH 10 [42]. Although these molecular changes in GA are reversible at physiological pH (to convert it back to its carboxylate monoanion, H3GA^−^), the partial loss of the antioxidant activity of GA released in the AKP fraction is possible. Lastly, the IC_50_ values for *p*-coumaric and 2-hydroxybenzoic acids were not reached at any of the assayed concentrations; the number and position of the OH- groups seem to have a substantial impact on DPPH scavenging.

FRAP assay was selected to evaluate the ability of PP and the extract’s fractions to stabilize radicals through SET mechanisms. GA was by far the most efficient antioxidant (0.0329 ± 2.432 × 10^−6^ µmoles TE/moles of GA) relative to all other pure PP, including mangiferin (0.0028 ± 2.487 × 10^−7^ µmoles TE/moles of antioxidant) and AKP (0.0047 ± 2.132 × 10^−7^ µmoles/g). In addition to the apparently scarce reactivity of 2-hydroxybenzoic and *p*-coumaric acids, which had the poorest activity in DPPH, and FRAP assays, ellagic acid, FP and AP fractions were also poorly effective compared to the other antioxidant values by this method. The most reasonable explanation for this finding are diverse synergistic/antagonistic actions between PP, resulting in reduced antioxidant activities for both the FP and AP fractions [42].

Lastly, ORAC assay was performed to test the antioxidant activity of monomer PP standards and extract’s fractions through HAT mechanism. According to our results, protocatechuic, 2-hydroxybenzoic, *p*-coumaric acids, and catechin were the most effective antioxidants (around 6400 to 7000 mmoles TE/moles of antioxidant) in this assay; all of them were present in trace amounts in AKP, and were not found in other extract’s fraction. Since these three phenolic acids are more likely to be absorbed in the intestine (>82%), according to chemoinformatic results further discussed, than GA (which showed a low ORAC value), they can have different bioactivities within the small intestine if they are released in vivo.

### 2.3. Antiproliferative Activity of Mango “Ataulfo” Peel Polyphenols

**Figure 1:**
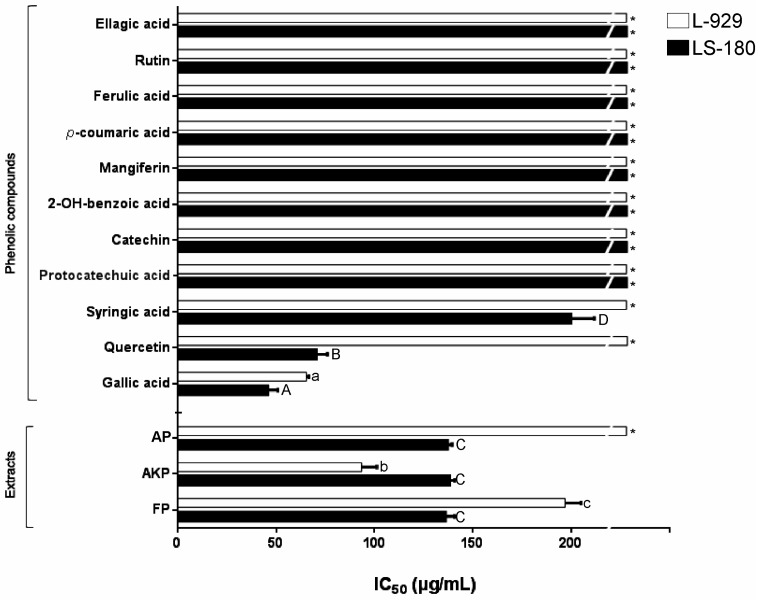
Antiproliferative activity of ‘Ataulfo’ mango peel polyphenol extract’s fractions and their main species contribution in two cell lines. Human colon cancer (LS180, ATCC^®^ CL-187™) and normal mouse subcutaneous connective tissue (L929; CCL-1TM). Results are expressed as mean ± standard deviation (*n* = 3). Different letters within bars indicated significant differences. Lower-case letters indicate differences among L929 treatments, whereas upper-case letters are used for differences among LS180 cell line treatments (*p* < 0.05). * IC_50_ > 200 µg/mL.

**Figure 2:**
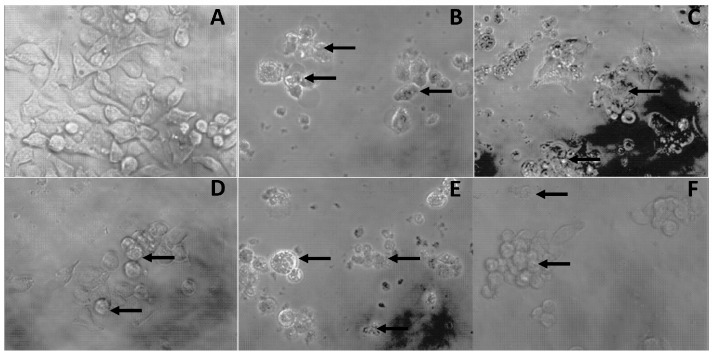
Pro-apoptotic events after 48 h in LS180 cells. Control cells (**A**); treated with gallic acid (**B**); quercetin (**C**); free phenolic extract (**D**); alkaline-treated fraction (**E**); or acid-treated fraction (**F**). Photomicrographs were taken at 100× magnification. Arrows indicate signs of cell blebbing or shrinkage.

The antiproliferative effects of PP depend on their molecular structures, and are often expressed as the minimum concentration that is required to inhibit the growth of cancer cells by 50% (IC_50_) [33,43]. The antiproliferative capacity, and pro-apoptotic effect of monomer PP standards and extract’s fractions are depicted in Figure 1 and Figure 2, respectively. Although three fractions were moderately effective against LS180 cells (~137 µg/mL), FP and AKP inhibited the growth of normal L929 cells (~197 and ~94 µg/mL, respectively). Ali, et al. [44] reported an IC_50_ > 250 µg/mL for an 80% ethanol extract obtained from ripe ‘Irwin’ mango peel on human gastric cancer (AGS), human cervical cancer (HeLa), and human hepatocarcinoma (HepG2) cells; however, Luo, Fu, Xiang, Yan, Hu, Huang, Huang, Sun, Li, and Chen [11] found an IC_50_ > 1000 µg/mL for the acetone extracts, which are rich in flavonoids (fisetin), and carotenoids, obtained from ‘Big Tainong’, ‘Small Tainong’, ‘Egg’, and ‘Australian’ mango peels. Subramanian, et al. [45] reported that the percent of viable HeLa cells remaining after treatment with 80% acetonic extracts (200 μg/ mL) from five different Indian mango peels ranged from 33% (‘Fozli’) to 55% (‘Lakhna’). Benites Vílchez, et al. [46] reported that gallotannin-rich peel extracts (80% acetone) from three Chinese mango varieties inhibited the growth of human promyelocytic leukemia (HL-60) cells, estrogen negative breast cancer (MDA-MB-231), and HepG2 cells (IC_50_ 20–80 μg/mL). In almost all of these studies, the antiproliferative activities of the peel extracts were correlated with their antioxidant activities in a dose-dependent manner. However, raw peel extracts comprise a mixture of several PP and other bioactive molecules in such way that their IC_50_ values are often higher (less active) than that of monomeric PP standards.

Interestingly, GA was a more effective antioxidant than mangiferin, quercetin, or syringic acid, which seems to be related to its highest antiproliferative activity against LS180 (46 µg/mL) and L929 (65 µg/mL) cells to a lesser extent (Figure 1). As found in this study, García-Rivera, et al [47] reported that GA inhibited colon cancer cells (HCT-15) with an IC_50_ ~ 198 µg/mL, but was not reactive toward normal cells; the authors suggested that the associated mechanism was reactive oxygen species (ROS)-dependent apoptosis. Moreover, the high content of GA in the AKP fraction may explain its high antiproliferative effect on both cell lines. In a previous study, we reported the in vitro interactions of gallic acid with other phenolics and pectin, affecting the capacity to stabilize free radicals [48]. However, other type of molecular interaction and synergistic effects between GA and the other PP within AKP fraction may be occurring. These interactions may enhance the effect against chronic-degenerative diseases (CVD) or aging symptoms. In that sense, other studies such as the one performed by Davinelli, et al. [49], suggest that the combined action of equol and resveratrol may be considered as a safe and effective strategy to ameliorate discomfort in recently menopausal women by reducing its symptoms. In addition, PP combination may be an adequate strategy to trigger different responses related to cells protection. For example, Davinelli, et al. [50] provide evidence that epigallocatechin gallate (EGCG) and l-carnosine induce neuroprotective effects whilst increasing the viability of neuronal cells.

Our findings suggest a possible interaction among PP within AP fraction. As evidence, it is shown that quercetin had an effective and selective antiproliferative capacity, as it was cytotoxic on LS180 but not L929 cells. Furthermore, AP had low GA and higher quercetin content compared to AKP, thus the AP fraction and quercetin have the same effective and selective antiproliferative capacity. However, GA and its high antioxidant activity has been associated to many other benefits related to CVD. For example, Vimang^®^, an aqueous extract from mango stem bark rich in both mangiferin and GA, has analgesic, anti-inflammatory, antioxidant, and immune-suppressive actions but also antiproliferative activity against MDA-MB231 cells by inhibiting NFκB/DNA binding and NFκB translocation in the cell nucleus [11,51]. Olivas-Aguirre, González-Aguilar, Velderrain-Rodríguez, Torres-Moreno, Robles-Zepeda, Vázquez-Flores, Rosa, and Wall-Medrano [5] reported that penta-*O*-galloyl-glucoside inhibited the growth of MDA-MB-231 (33 μg/mL), HepG2 (8 μg/mL), and HL-60 (5 μg/mL) cells almost as well as GA (16, 6, and 2 μg/mL, respectively). Based on this evidence, GA, either bound or free, still has high antiproliferative activity and seems to be responsible for the antiproliferative capacity of mango “Ataulfo” peel polyphenols.

Apoptosis is an active physiological process resulting in cellular self-destruction, and is characterized by distinct morphologic changes, including cell shrinkage, membrane blebbing, chromatin condensation, and DNA fragmentation, as well as the formation of apoptotic bodies [44]. In this study, pro-apoptotic events (blebbing and/or shrinkage) were quite common in FP extract-, GA-, and quercetin-treated LS180 cells (Figure 2). We have recently reported such pro-apoptotic events in murine macrophages transformed by the Abelson murine leukemia virus (RAW 264.7) that were treated (200 µg/mL, 24–48 h) with an 80% methanol extract obtained from ‘Ataulfo’ mango kernel [44]. However, further studies should be carried out to elucidate the exact mechanism followed by either these extracts or the antioxidant molecules. For instance, another antiproliferative mechanism is the one proposed by Eghbaliferiz and Iranshahi [52] for the acetone extract of ‘Fozli’ mango peel toward HeLa cells, which is related to the proteolytic activation of caspases-3, -8, -9, and the degradation of poly ADP-ribose polymerase.

The cell-specific reactivity of mango peel’s PP deserves a special mention. According to Lozano, et al. [53], the pro-oxidant action of PP, flavonoids, anthocyanins, and carotenoids is typically catalyzed by transition metals such as Fe and Cu within cells under certain pH and O_2_ conditions. Eghbaliferiz and Iranshahi [52] suggested that the antioxidant/pro-oxidant reactions of catechins are responsible for their antiproliferative effects on HT29 cell lines, being the molecules associated with an efficient electron transfer capacity. Thus, small PP simply oxidized (e.g., GA and quercetin) can exhibit pro-oxidant activity, but bounded or polymerized PP (e.g., hydrolyzable phenols and proanthocyanidins) have little or no pro-oxidant properties [54]. Our results suggest that ‘Ataulfo’ peel PP, which function via SET mechanisms, can act as pro-oxidant compounds, and enhance ROS production to cytotoxic levels in both LS180 and L929 cells. However, further studies are needed to evaluate if synergistic SET effects may also be occurring. For instance, GA can stablish strong antagonistic (ellagic acid, catechin, and quercetin) and synergistic (chlorogenic, protocatechuic acids) antioxidant effects with other PP [55,56].

### 2.4. Cheminformatics of Mango Peel Polyphenols

According to the data shown in Table 3, the probability for enterocyte absorption of mango ‘Ataulfo’ peel PP is as follows: *p*-coumaric and 2-hydroxycinnamic acid (89%) > ferulic acid (86%) > syringic acid (83%) > protocatechuic acid (82) > GA (75) > catechin (71%) > quercetin (64%) > ellagic acid (60%) > mangiferina (40%) > rutin (16%). At least for GA and its derivatives, absorption percentages above 57% indicate they have good cellular permeability [57]; therefore, all compounds except for mangiferin, and rutin are considered potentially bioavailable to the enterocyte. In an earlier study [56], the apparent intestinal permeability coefficients (Papp) across the Caco-2/HT-29 monolayer of GA and GA coming from the same AKP reported here were much higher (2.48 and 2.61 × 10^−6^ cm/s) than that observed for FP and AP fractions and mangiferin; such cellular permeability was also related to a higher cellular antioxidant activity in the AKP fraction (51.6 ± 1.4%) when tested at 125 µg/mL. The absorption percentages of PP molecules within AKP and AP fractions, along with its higher cellular antioxidant activity and cellular permeability may be related to molecular pathways regulated by mitochondria. Recent studies had shown that individual or PP combined effects may improve mitochondrial functions by the attenuation of oxidative stress, the regulation of mitochondrial metabolism and biogenesis, and the modulation of cell-death signaling cascades, among other mitochondrial-independent effects [58,59,60].

Hence, mitochondrial-related PP’s antiproliferative mechanisms are not entirely attributed to their antioxidant activity, but to a combined effect from more than one triggered mechanism. As Gorlach, et al. [61] assert, beyond their antioxidant activity, some PP may decrease mitochondrial membrane fluidity or have a molecular mechanism related to: hexokinase inhibition, mimicking of the Bcl-2 homology-3 (BH3) domains, thiol redox inhibition, among others. Nevertheless, despite the fact that not all PP have the same properties and mitochondrial-related mechanisms, all of them have ROS-scavenging actions either at the ROS-removing or ROS-formation levels [62]. Furthermore, PP concentration and cellular environment may influence those actions, and whether or not PP act as pro-oxidant molecules. Although pro-oxidant PP selective antiproliferative effects are not fully known, it is attributed to the formation of a labile radical aroxil, or a labile redox complex with a metal cation promoted by pro-oxidant PP, as metal ions catalyze the ROS generation through Fenton or Fenton-like reactions [63].

The main conclusion was that GA, after its release from AKP, has a similar permeability and antioxidant activity as that observed for GA as free standard. Being cautious with anything inferred from this in silico simulation, good absorption of matrix-releasable PP can be expected. However, as shown in Table 1, most PP are bound, so their microbial degradation to free PP seems to be restricted to the large bowel. These facts may in turn help to explain the antiproliferative effects reported in Figure 2, and they suggest that this effect could be related to their permeability to a certain degree. By analyzing the absorption percentage (Table 2), specific antioxidant mechanism (Table 3), and specific antiproliferative effect (Figure 2) of these compounds, it seems very likely that PP functioning via an HAT mechanism are not as effective as those functioning via an SET mechanism in their inhibition of the viability of LS180 and L929 cells. Thus, PP associated to a higher electron transfer capacity, and its effectiveness as antiproliferative agents may be related to either pro-oxidant action or PP individual mitochondrial functions. Hence, even though molecules such as *p*-coumaric and 2-hydroxycinnamic acids have the highest absorption percentages and antioxidant activities in the ORAC assay, these molecules may not be acting as prooxidants and, therefore, are not enhancing ROS production in cells.

## 3. Materials and Methods

### 3.1. Chemicals and Standards

Pure standards (≥93%) of all reagents used were obtained from Sigma-Aldrich-Fluka (St. Louis, MO, USA). HPLC-grade solvents were obtained from JT-Baker (Mexico City, Mexico).

### 3.2. Mango Fruit Selection

Ripe ‘Ataulfo’ mango fruits were purchased in a local market in Hermosillo, Sonora, Mexico. Mango selection was carried out as described by Palafox-Carlos, Yahia, Islas-Osuna, Gutierrez-Martinez, Robles-Sánchez, and González-Aguilar [57] involving its size evaluation, color uniformity, and the absence of signs of deterioration. The selected fruits were transported immediately to the laboratory for analysis. All fruits were washed and peeled so the mango peels could be freeze-dried and stored at −80 °C (FreeZone 6 liter Benchtop Freeze Dry System; Labconco, Kansas City, MO, USA). Freeze-dried peels were ground with a blender (≤0.40 μm), packed in vacuum-sealed bags and kept at −20 °C until use.

### 3.3. Extraction of Mango Peel Polyphenols

Ground ‘Ataulfo’ mango peel was used in this study. Three different fractions of an extraction (FP, AKP-, and AP-hydrolyzed phenolics) were obtained by the method reported by Mattila and Kumpulainen [32] with minor modifications. The FP fraction was obtained from 0.5 g of sample that was dissolved in 7 mL of solution A (methanol (85 mL) + BHT (2 g/L) + 10% acetic acid (15 mL)), sonicated for 30 min, and diluted with distilled water (10 mL). The pH was then adjusted to 2.0, and 15 mL of solution B (diethyl ether/ethyl acetate (DE/EA, 1:1)) was added. After mild shaking, the organic phase was separated and dried overnight at room temperature.

Monomer PP released after alkaline- and acid-hydrolysis could then be obtained. The aqueous solution recovered from the FP fraction was further diluted with distilled water (12 mL), and 10 mL of 10 M NaOH was added. Alkaline hydrolysis was performed under anaerobic conditions by stirring the mixture overnight at room temperature using a magnetic stirrer. Monomer aglycone PP were then recovered by the same procedure using solution B in order to obtain AKP fraction. Thus, the remaining aqueous phase was then acidified with HCl (2.5 mL) and heated (85 °C) for 30 min. The mixture was cooled to room temperature and then processed in the same way as FP and AKP using solution B to obtain the AP fraction.

### 3.4. UPLC-DAD

The monomers (aglycone form) of polyphenols found within mango peel extract’s fractions were quantified using an UPLC™ system (Acquity, Waters Co., Milford, MA, USA) equipped with a photodiode array extended λ (PDA eλ) detector, an Acquity UPLC™ BEH C18 VanGuard precolumn (130 Å, 1.7 µm, 2.1 mm × 5 mm), and an UPLC™ BEH C18 column (1.7 µm, 3.0 × 100 mm) with a column temperature of 60 °C and an auto sampler set at 5 °C. Two solutions were used as mobile phases: water-0.5% formic acid (A) and 100% methanol (B). The flow and gradient changes are shown in the supplementary material (Table S1). The results are expressed as µg of PP/gram of dry weight (µg/g DW) using standard calibration curves. Those quantifiable compounds were selected as individual PP, and used as pure standards in antioxidant and antiproliferative assays.

### 3.5. Antioxidant Capacity

The antioxidant activity of the FP, AKP, and AP fractions and their identified released monomeric PP were evaluated by three methods. The radical scavenging activities based on DPPH (515 nm) and ferric reducing antioxidant power (FRAP; 630 nm) assays were determined using a spectrophotometer FLUOstar™ OMEGA (BMG LABTECH; Chicago, IL, USA) according to the procedures described by Palafox-Carlos, Yahia, Islas-Osuna, Gutierrez-Martinez, Robles-Sánchez, and González-Aguilar [57] and Benzie and Strain [64], respectively, but with minor modifications in reaction volumes as suggested by Palafox-Carlos, Yahia, Islas-Osuna, Gutierrez-Martinez, Robles-Sánchez, and González-Aguilar [57]. The percentage of inhibition of DPPH radical vs sample concentration (0–200 µg/mL) was plotted, and the effective concentration to reach 50% radical inhibition (EC50) was then calculated and expressed as µmoles of antioxidant/mL for individual PP assayed, and µg of extract/mL for FP, AKP, or AP fractions. FRAP values of individual PP were expressed as µmoles of trolox equivalents (TE)/moles of antioxidant, whereas values of extract’s fractions were expressed in µmoles TE/g of extract (µmoles/g). The ORAC assay was performed according to Ou, et al. [65], and the results for individual PP were expressed as millimoles TE/moles of antioxidant, and as µmoles/g of dried extract for FP, AKP, and AP extract’s fractions. All assays were carried out according to the conditions recently reported by our group [66].

### 3.6. Cell Lines and Culture

Human colon adenocarcinoma (LS180, ATCC^®^ CL-187™) and normal mouse subcutaneous connective tissue (L929; CCL-1TM) cell lines were obtained from the American Type Culture Collection (ATCC^®^, Rockville, MD, USA). These cells were cultured in DMEM/5% FBS/penicillin (100 U/mL)/L-arginine + L-asparagine + L-glutamine + sodium pyruvate in 25 cm^2^ culture dishes under standard conditions (5% CO_2_, 37 °C, and 95% relative humidity).

### 3.7. Antiproliferative Activity (MTT Assay)

The antiproliferative activity of DMSO-dissolved extract’s fractions was evaluated by the MTT reduction assay following a previously reported procedure [67]. Briefly, a cell suspension (2 × 105 cells/mL) was placed in 96-well Corning Costar ^®^ culture plates and incubated for 24 h. Commercial standard PP and the extract’s fractions (FP, AKP, and AP) were dissolved at a non-toxic final DMSO concentration (0.25%). Cells were then treated with different concentrations of the extract’s fractions for 48 h. After the 48 h, cells were washed with DMEM/5% FBS to subsequently add 10 µL of MTT (5 mg/mL) and incubated for an additional 4 h. After the second incubation period, the formazan crystals were dissolved in acidified isopropanol, and their absorbance (570 and 630 nm) was measured using a microplate reader (iMark™; Bio-Rad, Hercules, CA, USA). The results were expressed as IC_50_ values (µg/mL). Finally, pro-apoptotic effects in LS180 cells between 12–24 h were observed by inverted microscopy at 40× magnification [5].

### 3.8. Cheminformatics

Chemical structures and SMILES (simplified molecular-input line-entry system) codes of all the monomers PP identified by UPLC-DAD were obtained from the PubChem Open Chemistry Database (https://pubchem.ncbi.nlm.nih.gov/search/). Relevant molecular features related to their enteral absorption capacity (molecular weight (MW; g/mol), total polar surface area (TPSA), octanol/water partition coefficient (LogPo/w), Lipinski’s rule of five (LIRF) and theoretical percentage of absorption (% Abs)) were further obtained by using the Molinspiration^©^ Cheminformatics software (http://www.molinspiration.com/) as described by Ertl and Schuffenhauer [68].

### 3.9. Statistical Analysis

A completely random design was applied to all experiments in this study, where all data was expressed as mean ± standard deviation (*n* = 3). Statistical differences among treatments were analyzed by one-way ANOVA and Tukey–Kramer multiple comparison test (*p* < 0.05) using the statistical software NCSS 2007.

## 4. Conclusions

Results from this study indicate that mango ‘Ataulfo’ peel is a promising source of PP (free and bound) with antiproliferative capacity useful to prepare plant-based over-the-counter nutritionals for the primary and secondary prevention of colon cancer. Particularly, our data suggests that GA is a major PP in this byproduct with antiproliferative capacity closely related to its in vitro antioxidant mechanism (SET), although other mechanisms could also be involved after its absorption in LS180 cells, as we previously reported that in Caco-2/HT-29 (75:25) the intracellular antioxidant activity of GA is preserved. We also do recognize that an in vitro antioxidant activity assay against free radicals of non-biological origin (e.g., DPPH or ABTS) represents a non-specific biological defense mechanism in cells, as the PP mechanisms that modulate cells’ redox behavior are not known yet. However, the evidence showed that the higher GA content in mango extract and its fractions, along with GA antioxidant mechanism, specifically the electron transfer, apparently are related to the observed antiproliferative effect.

## Figures and Tables

**Table 1:** Monomeric polyphenols (µg/g dry weight) in ‘Ataulfo’ mango peel extracts.

Compound Name	FP	AKP	AP
Mangiferin	1259 ± 105 ^a,A^	967 ± 20 ^b,B^	157 ± 52 ^b,C^
Gallic acid	--	23,816 ± 284 ^a,A^	5610 ± 8 ^a,B^
Quercetin	--	33 ± 3 ^d,A^	51 ± 5 ^c,B^
Catechin	75 ± 9 ^b,A^	60 ± 8 ^d,A^	--
Syringic acid	19 ± 2 ^b^	--	--
*p*-Coumaric acid	--	202 ± 13 ^c,d^	--
2-Hydroxybenzoic acid	--	700 ± 7 ^b^	--
Ferulic acid	--	12 ± 1 ^d^	--
Protocatechuic acid	--	129 ± 1 ^c,d^	--
Rutin	--	390 ± 11 ^c^	--
Ellagic acid	--	--	29 ± 9 ^c^

Values are expressed as the mean ± standard deviation (*n* = 3). Different lower-case letters within a column indicates significant differences among polyphenols content within a specific extract’s fraction, whereas the uppercase letters indicate differences between the content among the different fractions (*p* < 0.05). Below quantification limit (--), free phenolics (FP) and alkaline (AKP)- and acid (AP)-releasable phenolics.

**Table 2:** Antioxidant activity of and ‘Ataulfo’ mango peel polyphenol extract’s fractions and individual standards.

Fraction/PP Standard	DPPH *	FRAP *	ORAC
FP	40,200 ± 0.004 ^C^	20 ± 0.001 ^C^	1 ± 0.000 * ^A^
AKP	22,510 ± 0.109 ^A^	47 ± 0.002 ^A^	3 ± 0.000 * ^C^
AP	35,000 ± 30 ^B^	26± 0.009 ^B^	2 ± 0.000 * ^B^
Mangiferin	30 ± 1.000 ^b^	20 ± 0.002 ^d^	3.765 ± 256.691 ^d,e^
Gallic acid	140 ± 6.000 ^a^	320 ± 0.024 ^a^	1.105 ± 112.510 ^a^
Quercetin	160 ± 0.001 ^a^	50 ± 3.539 ^e^	5.177 ± 0.233 ^f^
Catechin	160 ± 0.004 ^a^	30 ± 6.119 ^b,d^	6.918 ± 0.167 ^h,g^
Syringic acid	230 ± 0.001 ^c^	50 ± 1.140 ^b,d^	2.394 ± 0.103 ^c^
*p*-Coumaric acid	--	0.211 ± 0.008 ^c^	3.639 ± 0.085 ^d^
2-Hydroxybenzoic acid	--	0.022 ± 0.000 ^c^	4.200 ± 0.071 ^e^
Ferulic acid	1100 ± 0.002 ^d^	30 ± 3.939 ^b^	3.527 ± 0.056 ^d^
Protocatechuic acid	280 ± 0.007 ^b^	50 ± 4.858 ^b,d^	6.459 ± 0.058 ^g^
Rutin	650 ± 0.001 ^e^	10 ± 1.116 ^b,d^	6.410 ± 0.305 ^g^
Ellagic acid	120 ± 0.001 ^a^	1 ± 0.121 ^c^	1.762 ± 0.115 ^b^

Individual phenols antioxidant values are expressed as µmoles of antioxidant/ mL (IC_50_ DPPH), µmoles TE/moles of antioxidant (FRAP) and moles TE/moles of antioxidant (ORAC). Extract fractions antioxidant values are expressed as µg of extract/mL (IC_50_ DPPH), µmoles TE/g of extract (FRAP, ORAC). Polyphenol (PP), non-detected (--), phenolics (FP) and alkaline (AKP) and acid (AP)-hydrolyzed phenolics (stepwise fractionation). Single asterisk (*) equals to row or column values multiplied by 10^−4^. Values are expressed as the mean ± standard deviation (*n* = 3): Lower-case letters were used to indicate significant differences (*p* < 0.05) among individual polyphenols, whereas upper-case letters were used to indicate significant differences among different extract’s fractions.

**Table 3:** Cheminformatics * of major polyphenol molecules from ‘Ataulfo’ mango peel.

Compound Name	MW	TPSA	logPo/w	LIRF	% Absorption
Mangiferin	422.3	201.3	−0.16	2	39.6
Gallic acid	170.1	98.0	0.59	0	75.2
Quercetin	302.2	131.4	1.68	0	63.6
Catechin	290.3	110.4	1.37	0	70.9
Syringic acid	198.2	76.0	1.20	0	82.8
*p*-Coumaric acid	164.2	57.5	1.43	0	89.2
2-Hydroxybenzoic acid	138.1	57.5	1.87	0	89.2
Ferulic acid	194.2	66.8	1.25	0	86.0
Protocatechuic acid	154.1	77.8	0.88	0	82.2
Rutin	610.5	269.4	−1.06	3	16.0
Ellagic acid	302.2	141.3	0.94	0	60.2

* Molinspiration Cheminformatics (http://www.molinspiration.com/). Molecular weight (MW; g/mol), total polar surface area (TPSA), octanol/water partition coefficient (logPo/w), Lipinski’s rule of five (LIRF).

## Supplementary Materials

Supplementary materials are available online. Table S1 Flow and gradient changes for phenolic compounds quantification by UPLC-DAD method.
