# Supplementary material for: Gallic Acid Content and an Antioxidant Mechanism Are Responsible for the Antiproliferative Activity of ‘Ataulfo’ Mango Peel on LS180 Cells
Source: Molecules. 2018 Mar 19;23(3):695. doi: 10.3390/molecules23030695 (PMC6017175; doi:10.3390/molecules23030695)
Supplement: Supplementary file 1 [file molecules-23-00695-s001.pdf]

Table S1. Flow and gradient changes for phenolic compounds quantification by UPLC-DAD method.

Time	Flow (mL/min)	%A	%B	Curve
Initial	0.4	80.0	20.0	Initial
0.25	0.150	80.0	20.0	6
5.00	0.200	80.0	20.0	6
12.00	0.180	55.0	45.0	5
25.00	0.100	0.0	100.0	5
26.00	0.200	60.0	40.0	5
27.00	0.400	80.0	20.0	6
30.00	0.400	80.0	20.0	6

A= Water-0.1% formic acid; B= Methanol
